# The Influence of Powder Composition and Hydrogen Consumption on the Structural, Corrosion and Tribological Characteristics of Fe-Cr-Al Coatings Obtained by Air Plasma Spraying

**DOI:** 10.3390/ma18235395

**Published:** 2025-11-29

**Authors:** Aidar Kengesbekov, Dastan Buitkenov, Garip Erdogan, Aiym Nabioldina, Sultan Komekov

**Affiliations:** 1 Institute of Composite Materials, Ust-Kamenogorsk 070000, Kazakhstan; kenesbekovaidar@gmail.com (A.K.); s.komekov.dodo@gmail.com (S.K.); 2 Research Center Surface Engineering and Tribology, Sarsen Amanzholov East Kazakhstan University, Ust-Kamenogorsk 070000, Kazakhstan; dbuitkenov@vku.edu.kz; 3 Metallurgical and Materials Engineering Department, Sakarya University, 54050 Sakarya, Turkey; gerdogan@sakarya.edu.tr

**Keywords:** Fe-Cr-Al coatings, air plasma spraying (APS), hydrogen flow rate, microstructure, X-ray phase analysis, corrosion resistance, friction coefficient

## Abstract

Fe-Cr-Al coatings were obtained by air plasma spraying (APS) from 85Fe-12Cr-3Al and 68Fe-26Cr-6Al powders at two hydrogen flow rates (8 and 13 L/min), which resulted in four deposition regimes (A1, A2, B1, B2). Stainless steel 20Kh13 (equivalent to AISI 420) was used as the substrate material. The microstructure of the coatings has a typical lamellar layering with molten and semi-molten particles. When the hydrogen flow rate is increased to 13 L/min, a denser and more homogeneous structure with reduced porosity is observed. X-ray phase analysis revealed the presence of metal and oxide phases (Fe,Cr), Fe_3_O_4_, FeO, Fe^2+^Cr_2_O_4_, which indicates partial oxidation of particles during the spraying process and stabilization of the structure. Electrochemical tests in 3.5% NaCl solution showed that the 85Fe-12Cr-3Al coatings are characterized by a corrosion potential of E_o_ ≈ −0.60…−0.67 V, a corrosion current density of i_o_ = (2.6–4.7) × 10^−5^ A/cm^2^, and a corrosion rate of 0.30–0.55 mm/year, whereas the 68Fe-26Cr-6Al coatings exhibit lower values of i_o_ = (1.4–2.9) × 10^−5^ A/cm^2^ and a corrosion rate of 0.17–0.34 mm/year, indicating the formation of a denser protective oxide film (Cr_2_O_3_ + Al_2_O_3_) and enhanced surface passivation. Tribological tests showed that 85Fe-12Cr-3Al coatings demonstrate more stable friction compared to 68Fe-26Cr-6Al, while for regime B2, after 180 m, an increase in the friction coefficient is observed, caused by brittleness and the local destruction of the oxide film. A comprehensive analysis of the results showed that increasing the hydrogen consumption to 13 L/min improves the density and corrosion–tribological characteristics of the coatings.

## 1. Introduction

Modern high-temperature and aggressive operating environments impose increasingly stringent requirements on the durability and reliability of structural materials. This problem is particularly acute in the energy sector, aviation and gas turbines, as well as in nuclear technology, where components are exposed to high temperatures, oxidizing gas environments, cyclic stresses, and chemically aggressive components [1,2,3,4,5]. In such conditions, standard stainless and heat-resistant steels may exhibit insufficient resistance to oxidation, scale formation, or corrosion under the influence of a gaseous medium. To increase their resistance, more and more attention is being paid to protective coatings based on Fe-Cr-Al systems, which form permanent oxide layers of α-Al_2_O_3_ and Cr_2_O_3_ upon heating, effectively preventing oxygen diffusion and slowing down the processes of high-temperature corrosion [6,7,8,9].

Fe-Cr-Al alloys have long established themselves as heat-resistant alloys used in heating elements, diffusion barriers, and heat-resistant structures due to their high resistance to oxidation and stability in the atmosphere [10,11,12]. In recent years, they have been actively investigated as candidates for accident-tolerant fuel (ATF) in nuclear reactors, such as fuel element shells or protective coatings on zirconium alloys (Zr alloys) [13,14,15]. Due to the formation of slow-growing Al_2_O_3_ oxide, FeCrAl coatings demonstrate high resistance to oxidation and steam corrosion under emergency conditions (Loss of Coolant Accident, LOCA) [16,17]. In addition, these alloys are considered as potential materials for structural elements and the first stand in fissionable and thermonuclear installations. In gas turbine installations, FeCrAl alloys are used as bonding (bond coat) layers in thermal barrier systems, as well as permanent heat-resistant coatings for blades and combustion chambers [18,19,20]. In the chemical and oil and gas industries, they are used as catalyst carriers and protective barriers resistant to sulfur-containing and hydrocarbon media [21,22]. In addition, FeCrAl coatings show excellent corrosion resistance in contact with liquid metals and melts (for example, Pb-Bi or Na), which makes them promising for heat exchange systems and reactors with liquid–metal coolant [23,24].

The properties of Fe-Cr-Al alloys are largely determined by the ratio of iron, chromium, and aluminum, which affect the microstructure, phase stability, and kinetics of the formation of protective oxide films. An increase in the content of Cr and Al contributes to the formation of dense and thermally stable α-Al_2_O_3_ layers, but excessive alloying may increase brittleness and internal stresses, while an increased content of Fe improves the adhesion and ductility of the coating, but reduces oxidation stability [25,26,27]. According to [23], alloys with 3–7 wt.% Al forms a stable α-Al_2_O_3_ layer, whereas with a decrease in the proportion of aluminum, unstable oxides (Fe,Cr)_3_O_4_ are formed. Studies of Fe-xCr-6Al systems with a change in chromium content from 10 to 25 wt.% showed an improvement in the structural stability and heat resistance of coatings compared to alloys with lower chromium content, due to the formation of denser Cr_2_O_3_ and Al_2_O_3_ phases [28].

Along with heat and oxidation resistance, the mechanical, tribological and corrosion properties of FeCrAl coatings are important operational characteristics. It was shown in [29] that coatings obtained by air plasma spraying (APS) have a microhardness of 4–5 GPa and a coefficient of friction in the range of 0.6–0.8, depending on the spraying regimes and the microstructure of the coating. An increase in the content of Cr and Al leads to the formation of denser oxide phases and a decrease in the wear rate, which is associated with an increase in hardness and stabilization of oxide films. According to data from [30], heat treatment helps to compact the structure, reduce porosity, and increase the adhesive strength of the coating. The study [31] noted that FeCrAl coatings modified with WC and TiC carbides exhibit increased microhardness, improved tribological characteristics, and high resistance to adhesive and abrasive wear.

Based on these studies, the choice of formulations 85Fe-12Cr-3Al and 68Fe-26Cr-6Al seems scientifically sound and relevant. The first composition is characterized by a high iron content, which ensures good adhesion to the substrate and high plasticity. The second composition contains increased proportions of chromium and aluminum, which contributes to the formation of dense and stable oxide phases α-Al_2_O_3_ and Cr_2_O_3_, which enhance the protective properties of the coating. Their comparative study makes it possible to establish the relationship between the chemical composition, microstructure, mechanical, tribological and corrosion characteristics of coatings, which is especially important for increasing the durability of steels used in energy and mechanical engineering. It should be noted that despite a significant amount of work devoted to the structure and properties of Fe-Cr-Al coatings, the effect of air plasma spraying parameters, in particular changes in hydrogen consumption in the plasma-forming gas mixture, on the formation of microstructure, phase composition, corrosion and tribological characteristics remains insufficiently studied. Meanwhile, hydrogen has a significant effect on the temperature of the plasma jet, the degree of melting of powder particles and their cooling rate, which directly determines the morphology and density of the coating.

In this regard, the purpose of this work is to obtain and comparatively analyze 85Fe-12Cr-3Al and 68Fe-26Cr-6Al coatings deposited by air plasma spraying with different hydrogen contents in the plasma-forming mixture on a 20Kh13 steel substrate, with the determination of their microstructure, phase composition, mechanical, tribological and corrosion properties.

## 2. Materials and Methods

The coatings of the Fe-Cr-Al system were obtained by air plasma spraying (APS) using an F4-MB type installation (Sulzer Metco, Winterhur, Switzerland) under constant technological conditions shown in Table 1. In the experiment, two types of powders and two levels of hydrogen consumption in the plasma-forming mixture (8 and 13 L/min) were used, which ensured the formation of four spraying regimes. For convenience of analysis and interpretation of the results, the samples were conventionally designated as A1 (85Fe12Cr3Al, 8 L/min H_2_), A2 (85Fe12Cr3Al, 13 L/min H_2_), B1 (68Fe-26Cr-6Al, 8 L/min H_2_) and B2 (68Fe-26Cr-6Al, 13 L/min H_2_), where letters A and B correspond to the composition of the powders, and the numbers indicate the consumption of hydrogen in the plasma-forming mixture.

The SprayWatch 2s system, equipped with a high-speed camera and optical sensors, was used to monitor the characteristics of the plasma plume during spraying. The system recorded the temperature and velocity of the particles in real time, as well as the parameters of the jet–width, angle of divergence and density of the powder flow.

Stainless steel, grade 20Kh13 (analogous to AISI 420), was used as the substrate material. Prior to coating application, the surface of the samples (40 × 40 × 4 mm) was degreased in acetone and subjected to sandblasting to enhance adhesion between the coating and the substrate.

**Figure 1:**
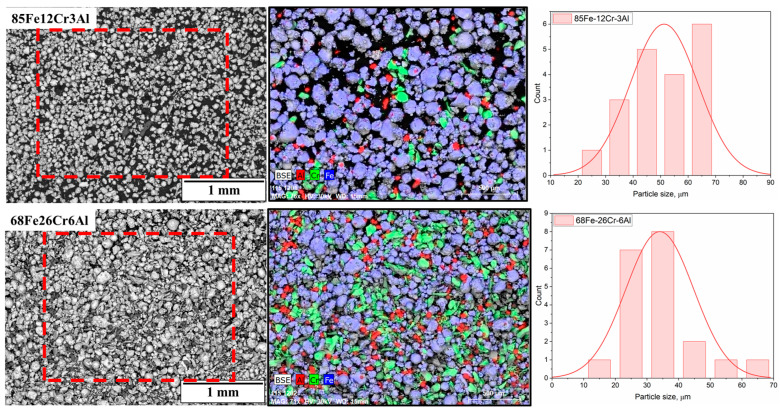
SEM images of 85Fe-12Cr-3Al (top row) and 68Fe-26Cr-6Al (bottom row) alloy powders, corresponding EDS elemental maps for Fe, Cr, and Al and particle size distributions.

As the starting materials, powders of pure metals—iron (Fe), chromium (Cr), and aluminum (Al)—with a chemical purity of no less than 98% and particle sizes ranging from 15 to 40 µm were used. To obtain powder mixtures with compositions of 85Fe-12Cr-3Al and 68Fe-26Cr-6Al (wt.%), the corresponding amounts of elemental powders were mechanically mixed in an Emax planetary ball mill for 4 h at a rotation speed of 200 rpm. Figure 1 presents the morphology, elemental composition, and particle size distribution of the 85Fe-12Cr-3Al and 68Fe-26Cr-6Al powder mixtures obtained by mechanical mixing of pure metal powders (Fe, Cr, Al) in a ball mill. The SEM images (BSE mode) show a wide range of particle shapes and sizes in both compositions. The particles predominantly exhibit irregular shapes, which is typical for powders subjected to intensive mechanical treatment. The EDS elemental maps reveal the presence of individual particles enriched in iron (blue), chromium (green), and aluminum (red), which is attributed to the absence of preliminary alloying. This pattern reflects physical mixing rather than metallurgical bonding of the components and indicates that the mixtures retain a heterogeneous structure at this stage. However, the particles are uniformly distributed across the field of view without signs of agglomeration or macroscopic segregation.

The microstructure of the coatings was studied using a scanning electron microscope (SEM, TESCAN Vega 4, Tescan, Brno, CzechRepublic) in the mode of back-reflected electrons. The phase composition of the coatings was determined by X-ray phase analysis (XRD) on an X-Pert Pro diffractometer, under CuKa radiation (λ = 1.5406 Å). Phase identification was carried out using the JCPDS database, and the processing and decoding of diffractograms was performed using the MDI JADE 6.5 software.

The electrochemical corrosion characteristics of the coated samples were studied by the method of potentiodynamic polarization. An aqueous solution of 0.5 M NaCl was used as a corrosive medium. The tests were carried out in a Gamry Paracell three-electrode cell, where a coating sample was used as a working electrode, a graphite electrode was used as an auxiliary electrode, and a saturated calomel electrode served as a reference electrode. The size of the studied samples was 10 × 10 mm, while the area in contact with the electrolyte was limited to 0.28 cm^2^. Before each series of measurements, the open circuit potential was recorded for 30 min until the stationary value of the corrosion potential was reached. The potentiodynamic polarization was performed at a scan rate of 1 mV/s.

Tribological coating tests were performed using a TRB^3^ tribometer (Anton Paar, Graz, Austria) according to the ball-on-disk scheme in the “one-way pass” sliding mode. A ball of 100Cr6 with a diameter of 6 mm was used as a counterbody. The tests were performed at a normal load of 5 N, a linear velocity of 10 cm/s and a friction path length of 300 m. The track radius was 5 mm. The tests were carried out at room temperature in air without lubrication.

## 3. Results

To establish the relationship between the technological parameters and the stability of the air plasma spraying process, the characteristics of the plasma torch were monitored using the SprayWatch 2s system, which provides real-time measurement of paritcle temperature (SprayTemp), particle velocity, particle flux density, jet Width, and jet divergence and standard deviations (STD), characterizing the stability of the process. In regime A1 (85Fe12Cr3Al, 8 L/min H_2_), the particle temperature was 2550–2650 °C, the particle velocity was 203–227 m/s, and the jet divergence stabilized at 5.3 °C, having decreased from an initial value of 6.0 °C. This indicates arc stabilization and uniform particle transport. With an increase in the hydrogen flow rate to 13 L/min (regime A2), the particle temperature increased to 2760–2850 °C, the particle velocity to 210–220 m/s, while the standard deviation of temperature decreased to 5–14 °C, and the divergence of the jet was about 4.5°, which indicates the formation of a stable and focused flow. In the regime B1, a temperature of about 2880 °C and a velocity of 206–223 m/s were recorded with a jet width of 12–15 mm and a divergence of 3.2–4.9 °C. After a short transition period, the standard deviations of temperature and velocity decreased to minimum values (Spray-TempStd = 2–5 °C, VelocitySTD = 2–3 m/s), indicating high stability process and energy-saturated torch with uniform melting of powder. For the B2 regime, the particle temperature was in the range of 2600–2720 °C, the particle velocity was 186–225 m/s, the jet width was about 17.8 mm, and the divergence was 4.6–5.8 °C. Despite stable temperature and velocity values (Spray-TempSTD ≤ 12 °C, VelocitySTD~2–4 m/s), significant fluctuations in the powder flow (FluxSTD = 8–21) were observed, indicating uneven material supply.

From a physico-chemical point of view, an increase in hydrogen consumption changes the thermal and electrical characteristics of the plasma arc. Hydrogen, having high thermal conductivity and low molecular weight, increases the efficiency of heat transfer and contributes to arc compression. The processes of dissociation and recombination of H_2_–2H are accompanied by the release of heat, which increases the enthalpy and energy intensity of the plasma. As a result, the divergence angle of the jet decreases and the uniformity of the temperature distribution in the axial direction increases, which improves the transfer and melting of powder particles. Additionally, it was established that the chemical composition of the powders also affects the characteristics of the plasma torch. During the transition from alloy 85Fe-12Cr-3Al to 68Fe-26Cr-6Al, a moderate decrease in temperature and particle velocity was observed with a decrease in standard deviations, which may be due to the higher thermal conductivity and heat capacity of alloy 68Fe-26Cr-6Al, contributing to arc stabilization and smoothing temperature fluctuations.

**Figure 2:**
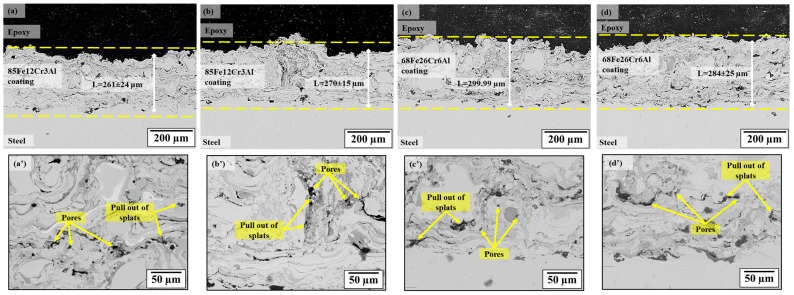
Cross-sections of Fe-Cr-Al coatings obtained under various APS regimes: (**a**,**a’**) regime A1, (**b**,**b’**) regime A2, (**c**,**c’**) regime B1, (**d**,**d’**) regime B2.

Figure 2 shows the cross-sections of the Fe-Cr-Al coating obtained under four regimes of air plasma spraying corresponding to different hydrogen consumption and chemical composition of the powders. For all regimes, a typical lamellar structure is observed, formed by the deposition of molten particles (splats), as well as characteristic interlamellar pores and oxide layers. In regime A1, at a relatively low particle temperature (2250–2650 °C) and particle velocity (≈203–227 m/s), the microstructure of the coating contains a significant number of non-molten and semi-molten particles, numerous interlamellar pores and areas of pull-out splats. This is due to the insufficient energy of the torch, at which individual particles do not have time to completely melt, and the sintering process on the substrate proceeds irregularly. The average porosity of the coating is 2.58%. With an increase in the hydrogen consumption to 13 L/min (regime A2), the temperature of the particles increases to 2760–2850 °C, which ensures more intensive melting of the powder and a reduction in defects. The coating acquires a denser and more uniform lamellar structure, and the porosity decreases to 2.18%.

The B1 coatings were formed at a higher particle temperature (≈2880 °C), but with an increased melt viscosity due to the high content of Cr and Al. As a result, the structure retains partially molten particles, individual gaps between the lamellae, and pores 1–5 microns in size, with a total porosity of 3.40%. An increase in hydrogen consumption to 13 L/min (B2) contributes to partial compaction of the structure; however, local areas of non-molten inclusions and small pores (≈3.14%) persist, indicating excessive turbulent mixing in the flow with increased heat transfer.

Figure 2a’–d’ shows enlarged fragments of the microstructure, where characteristic defects are clearly discernible–pores, pull-out of splats, as well as non-molten and semi-molten particles. Their number and distribution depend on the energy parameters of the plasma and the particle velocity determined during monitoring. In sample A1, where the temperature and velocity of the jet were the lowest, a high defect number was observed: interlamellar pores, incomplete fusion of lamellae. In regime A2, due to a more focused and energy-saturated flow (Divergence ≈ 4.5°, Spray-TempSTD < 10 °C), the structure becomes significantly denser, and the number of defects is reduced. 68Fe26Cr6Al alloys (regimes B1 and B2) are characterized by the presence of partially fused rounded particles and high microstructural heterogeneity, which is associated with the peculiarities of heat transfer of more fusible components and the high heat capacity of Fe-Cr-Al systems. That is, microstructural analysis, in conjunction with the results of monitoring the plasma jet parameters, confirms that an increase in hydrogen consumption leads to an increase in plasma enthalpy, better melting of powder particles, and a decrease in coating porosity. At the same time, the stability of temperature and velocity parameters directly determines the deposition of lamellae and a decrease in the number of defects.

**Figure 3:**
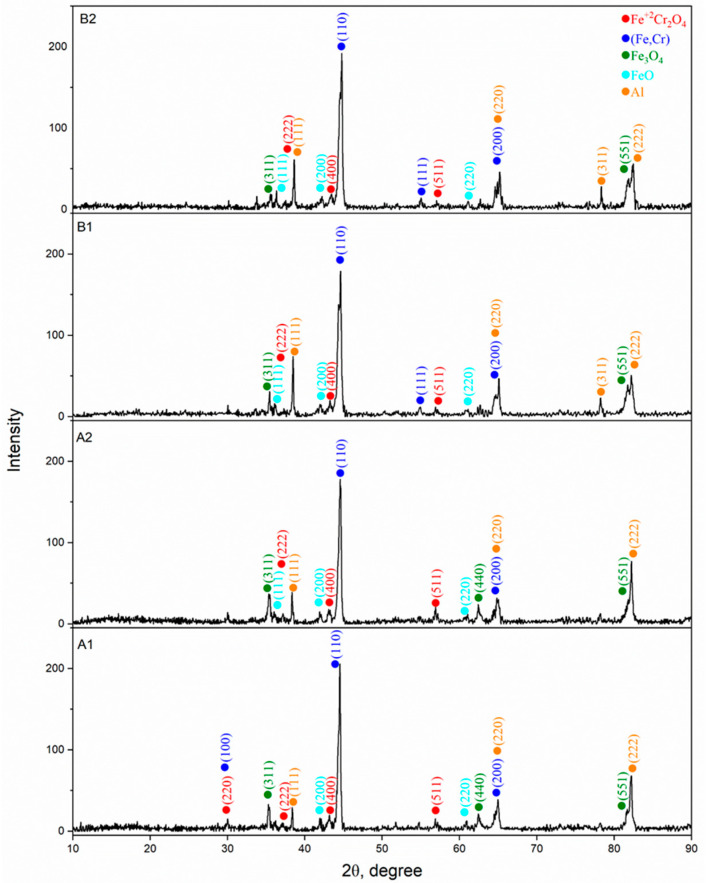
X-ray diffraction patterns of Fe-Cr-Al coatings obtained with various APS solutions.

X-ray phase analysis of coatings obtained under various regimes of air plasma spraying (Figure 3) showed the presence of several main phases in their composition: (Fe, Cr) a solid solution based on BCC-iron (ICDD 00-041-1466), Fe_3_O_4_ (ICDD 01-074-0748), FeO (ICDD 01-074-1880), Fe^2+^Cr_2_O_4_ (00-034-0140) and Al (01-085-1327) in the metallic state. The formation of such phases is a consequence of the partial oxidation of particles during spraying, as well as the interaction of iron, chromium and aluminum under conditions of rapid heating and cooling. In all coatings, the diffraction patterns are dominated by the peaks of the metallic (Fe,Cr) solid solution, indicating that the metallic matrix remains the primary structural component regardless of the spraying parameters. The oxide phases appear as weak reflections that are characteristic of thin oxide interlayers and interlamellar inclusions formed during APS as a result of partial in-flight oxidation of the particles. Comparison of the diffractograms of the A and B series shows that the phase composition is qualitatively identical in all cases, and the differences are expressed only in the relative intensities of individual reflections, which reflects the influence of plasma-jet parameters on particle melting and their interaction with atmospheric oxygen. The A-series coatings exhibit a combination of intense metallic peaks and weak oxide reflections, corresponding to a moderate degree of oxidation. The B-series coatings also contain oxide phases, although the relative intensities of their peaks vary with the spraying regime, which is associated with the thermal history of the particles at elevated Cr and Al content. Overall, the XRD results demonstrate that, regardless of the APS regime, the coatings are formed via a common mechanism involving a metallic (Fe,Cr) solid solution as the dominant phase and a limited amount of oxide phases, confirming the stability and reproducibility of the Fe-Cr-Al phase composition under varying spraying parameters.

**Figure 4:**
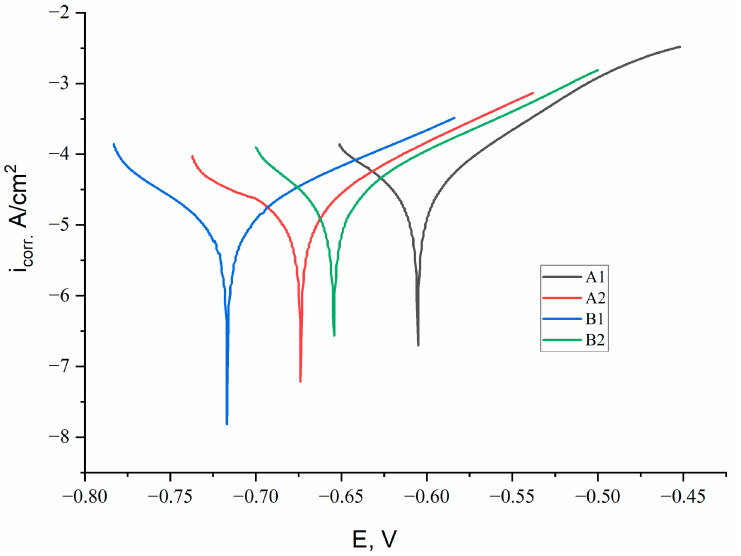
Potentiodynamic polarization curves of Fe-Cr-Al coatings obtained under various APS regimes.

Figure 4 shows the potentiodynamic polarization curves of the Fe-Cr-Al system coatings obtained under various APS regimes. The calculated electrochemical parameters–anode and cathode Tafel coefficients (β_a_, β_c_), corrosion potential (E_corr_), current density (i_corr_) and corrosion rate are summarized in Table 2.

According to the electrochemical parameters summarized in Table 2, the coatings produced under different APS regimes exhibit distinct corrosion behaviors. For sample A1, the anodic (β_a_ = 78.1 V/dec) and cathodic (β_c_ = 116.1 V/dec) Tafel slopes indicate relatively active anodic dissolution and moderately hindered cathodic reactions. The corrosion current density is the highest among all samples (i_corr_ = 4.67 × 10^−5^ A/cm^2^), while the corrosion potential (E_corr_ = −0.605 V) is shifted toward more negative values, reflecting lower thermodynamic stability. Consequently, the corrosion rate reaches 0.547 mpy, demonstrating the weakest corrosion resistance in this group. For sample A2, an increase in hydrogen flow rate leads to higher Tafel slopes (β_a_ = 93.6 V/dec, β_c_ = 172.0 V/dec), which reflects a slowdown of both anodic and cathodic processes. The corrosion current density decreases nearly twofold (i_corr_ = 2.58 × 10^−5^ A/cm^2^), and E_corr_ shifts slightly more negative (−0.674 V), which is typical for denser but chemically similar surfaces. The corrosion rate drops to 0.303 mpy, confirming that improved melting and densification of the lamellae enhance the protective properties of the coating. Sample B1 demonstrates the most favorable combination of electrochemical parameters. The anodic slope (β_a_ = 97.7 V/dec) suggests reduced dissolution kinetics, while the cathodic slope (β_c_ = 88.6 V/dec) indicates relatively slow cathodic reactions. This coating exhibits the lowest corrosion current density (i_corr_ = 1.43 × 10^−5^ A/cm^2^) and the most positive corrosion potential among all samples (E_corr_ = −0.717 V), reflecting high thermodynamic stability. The resulting corrosion rate is minimal (0.168 mpy), indicating that the higher Cr and Al content in the 68Fe-26Cr-6Al composition effectively promotes the formation of a stable passive oxide layer. For sample B2, the anodic and cathodic slopes (β_a_ = 90.2 V/dec and β_c_ = 82.7 V/dec, respectively) indicate moderate inhibition of both anodic and cathodic processes. The corrosion current density is slightly higher than that of B1 (i_corr_ = 2.90 × 10^−5^ A/cm^2^), and the corrosion potential shifts to −0.654 V. The corresponding corrosion rate is 0.340 mpy, which is lower than that of both A1 and A2 but higher than B1. This behavior reflects a balance between improved lamellar compaction and localized defects formed at higher hydrogen flow rates. most optimal combination of kinetic and thermodynamic electrochemical parameters.

**Figure 5:**
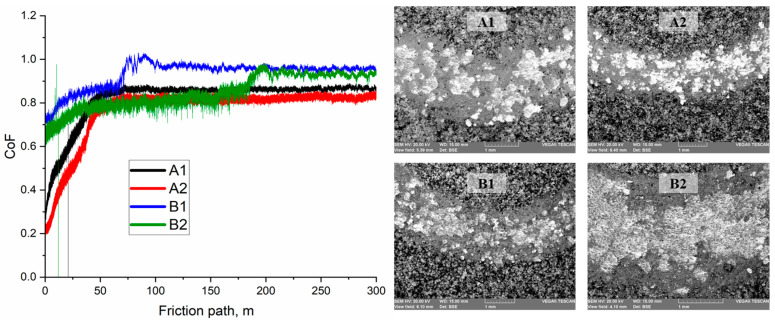
Dependence of the coefficient of friction (µ) on the friction path for Fe-Cr-Al coatings obtained under various APS regimes.

Figure 5 shows the dependences of the coefficient of friction (µ) on the friction path for coatings formed under various APS regimes. For all samples, a typical staged evolution of the curve is observed: in the initial section (up to 40–60 m), the friction coefficient increases due to the running-in of the counterbody and the removal of surface irregularities. After this stage, the value stabilizes with minor fluctuations. For the A1 coating, the friction coefficient stabilizes at 0.820 ± 0.107. It is assumed that the moderate friction stability may be related to the formation of an oxide film resulting from tribo-oxidation products. In regime A2, the average friction coefficient decreases to 0.764 ± 0.134. This is presumably due to a denser coating structure resulting from improved particle melting at a higher hydrogen flow rate. The curve exhibits a smooth increase in µ and a more uniform running-in process, which may indicate enhanced plasticity of the top layer. The B1 coating shows the most stable curve with minimal fluctuations and an average value of 0.933 ± 0.062. It can be assumed that the high friction stability is associated with the dense lamellar structure and the formation of a protective oxide film reducing micro-adhesion. For regime B2, the average friction coefficient was 0.849 ± 0.077. Up to 180 m, the curve remains stable; however, beyond this distance, a gradual increase in µ is observed. This may be associated with local damage to the surface oxide film and a transition to direct contact between lamellar structures. The increase in µ after 180 m may be caused by thermomechanical degradation of oxide regions and partial thinning of the protective layer, leading to an increase in the adhesive component of friction.

## 4. Conclusions

The following conclusions can be drawn from the data obtained:

1. Using the air plasma spraying method, Fe-Cr-Al coatings were obtained from 85Fe-12Cr-3Al and 68Fe-26Cr-6Al powders at a hydrogen flow rate of 8 and 13 L/min, which allowed the temperature and dynamics of the plasma jet to be varied.
2. It was established that an increase in H_2_ flow rate leads to an increase in particle temperature (up to 2850 °C) and particle velocity (up to 225 m/s), a decrease in the divergence angle, and an increase in process stability.
3. Coatings deposited at 13 L/min H_2_ have a denser, more uniform structure and minimal porosity, whereas at 8 L/min H_2_, areas with semi-molten particles and increased porosity are formed.
4. X-ray phase analysis revealed the presence of phases (Fe, Cr), Fe_3_O_4_, FeO, Fe^2+^Cr_2_O_4_ and Al, the formation of which is due to partial oxidation of particles during the spraying process.
5. Electrochemical tests in 3.5% NaCl showed that coatings of 68Fe-26Cr-6Al possess a lower corrosion current density (i_corr_ = (1.4–2.9) × 10^−5^ A/cm^2^) and corrosion rate (0.17–0.34 mm/year) compared with coatings of 85Fe-12Cr-3Al, for which i_corr_ = (2.6–4.7) × 10^−5^ A/cm^2^ and corrosion rate = 0.30–0.55 mm/year. This improvement is attributed to the formation of a stable protective oxide film consisting of Cr_2_O_3_ and Al_2_O_3_.
6. Tribological tests showed that 85Fe-12Cr-3Al coatings are characterized by more stable friction and smaller fluctuations in the friction coefficient (0.76–0.82) than 68Fe-26Cr-6Al coatings (0.85–0.93). In regime B2, an increase in the friction coefficient is observed after 180 m due to the destruction of the oxide layer.

Regime A2 (85Fe-12Cr-3Al, 13 L/min H_2_) is optimal in terms of structure, minimum porosity, corrosion resistance and tribological properties.

## Figures and Tables

**Table 1:** Coating spraying parameters.

Sample Name	Powder Composition	Powder Consumption, g/min	Argon Flow, L/min	Hydrogen Flow, L/min	Current, A
A1	85Fe12Cr3Al	28.5	45	8	500
A2	85Fe12Cr3Al	28.5	45	13	500
B1	68Fe-26Cr-6Al	27.6	45	8	500
B2	68Fe-26Cr-6Al	27.6	45	13	500

**Table 2:** Electrochemical parameters of Fe-Cr-Al coatings.

Samples	β_a_, V/dec	β_c_, V/dec	i_corr_, A/cm^2^	E_corr_, V	Corrosion Rate, mpy
A1	78.1	116.1	4.67 × 10^−5^	−0.605	0.547
A2	93.6	172.0	2.58 × 10^−5^	−0.674	0.303
B1	97.7	88.6	1.43 × 10^−5^	−0.717	0.168
B2	90.2	82.7	2.90 × 10^−5^	−0.654	0.340

## Data Availability

The original contributions presented in the study are included in the article, further inquiries can be directed to the corresponding author.

## References

[1] Cheng J., Dai Q., Lan W., Zhou X., Yu D. (2024). High-Temperature Corrosion Behavior of the FeCrAl Laser Cladding Coatings in Waste-to-Energy Superheaters: Influence of Al Content. Surf. Coat. Technol..

[2] Buitkenov D., Sagdoldina Z., Nabioldina A., Drenda C. (2025). The Study of Tribological Characteristics of YSZ/NiCrAlY Coatings and Their Resistance to CMAS at High Temperatures. Appl. Sci..

[3] Bolelli G., Hulka I., Koivuluoto H., Lusvarghi L., Milanti A., Niemi K., Vuoristo P. (2014). Properties of WC–FeCrAl Coatings Manufactured by Different High Velocity Thermal Spray Processes. Surf. Coat. Technol..

[4] Rakhadilov B., Buitkenov D., Apsezhanova A., Kakimzhanov D., Nabioldina A., Magazov N. (2025). Selection of Optimal Process Parameters for Arc Metallization. Coatings.

[5] Bolelli G., Colella A., Lusvarghi L., Puddu P., Rigon R., Sassatelli P., Testa V. (2019). Properties of HVOF-Sprayed TiC-FeCrAl Coatings. Wear.

[6] Steinbrueck M., Grosse M., Tang C., Stuckert J., Seifert H.J. (2024). An Overview of Mechanisms of the Degradation of Promising ATF Cladding Materials During Oxidation at High Temperatures. High Temp. Corros. Mater..

[7] Rakhadilov B., Sulyubayeva L., Maulet M., Sagdoldina Z., Buitkenov D., Issova A. (2024). Investigation of High-Temperature Oxidation of Homogeneous and Gradient Ni-Cr-Al Coatings Obtained by Detonation Spraying. Coatings.

[8] Dabney T., Johnson G., Yeom H., Maier B., Walters J., Sridharan K. (2019). Experimental Evaluation of Cold Spray FeCrAl Alloys Coated Zirconium-Alloy for Potential Accident Tolerant Fuel Cladding. Nucl. Mater. Energy.

[9] Kantay N., Kasmamytov N., Rakhadilov B., Plotnikov S., Paszkowski M., Kurbanbekov S. (2020). Influence of Temperature on Structural-Phase Changes and Physical Properties of Ceramics on the Basis of Aluminum Oxide and Silicon. Mater. Test..

[10] Qu H., Yin L., Larsen M., Rebak R.B. (2024). Distinctive Oxide Films Develop on the Surface of FeCrAl as the Environment Changes for Nuclear Fuel Cladding. Corros. Mater. Degrad..

[11] Qiao Y., Ni Y., Yang K., Wang P., Wang X., Liu R., Sun B., Bai C. (2025). Iron-Based High-Temperature Alloys: Alloying Strategies and New Opportunities. Materials.

[12] Wang J., Liu S., Bai X., Zhou X., Han X. (2020). Oxidation Behavior of Fe–Al–Cr Alloy at High Temperature: Experiment and a First Principle Study. Vacuum.

[13] Nagothi B.S., Qu H., Zhang W., Umretiya R.V., Dolley E., Rebak R.B. (2024). Hydrothermal Corrosion of Latest Generation of FeCrAl Alloys for Nuclear Fuel Cladding. Materials.

[14] Liao J., Wang H., Wu J., Zhang W., Xu F., Sun H., An X., Qiu S. (2022). Addition of Niobium in Fe-13Cr-4.5Al-2Mo Alloy Used as ATF Cladding: Effect on High Temperature Water Corrosion and in-Situ Electrochemistry. Mater. Des..

[15] Li N., Chen L.-Y., Wang Z.-X., Xuan H.-N., Chai L.-J., Yang H.-L., Oleksandr D., Lu S. (2025). Enhancement of Hardness and High-Temperature Oxidation Resistance of Cr/FeCrAl Dual-Layer Plasma-Sprayed Coating on Zr Substrate by Post-Processing. J. Mater. Res. Technol..

[16] Tang C., Jianu A., Steinbrueck M., Grosse M., Weisenburger A., Seifert H.J. (2018). Influence of Composition and Heating Schedules on Compatibility of FeCrAl Alloys with High-Temperature Steam. J. Nucl. Mater..

[17] Bunn J.K., Fang R.L., Albing M.R., Mehta A., Kramer M.J., Besser M.F., Hattrick-Simpers J.R. (2015). A High-Throughput Investigation of Fe–Cr–Al as a Novel High-Temperature Coating for Nuclear Cladding Materials. Nanotechnology.

[18] Gurevich L.M., Pronichev D.V., Slautin O.V., Tikhaeva V.V. (2023). Corrosion Resistance of Fe-Cr-Al Intermetallic Coatings Obtained by Aluminizing. Metals.

[19] Kulevich V.P., Slautin O.V., Kharlamov V.O. (2021). Evaluation of the Heat Resistance of the Fe-Cr-Al System Coatings. Defect Diffus. Forum.

[20] Leshchinsky E., Sobiesiak A., Maev R. (2018). Intermetallic Al-, Fe-, Co- and Ni-Based Thermal Barrier Coatings Prepared by Cold Spray for Applications on Low Heat Rejection Diesel Engines. J. Therm. Spray Technol..

[21] Pauletto G., Vaccari A., Groppi G., Bricaud L., Benito P., Boffito D.C., Lercher J.A., Patience G.S. (2020). FeCrAl as a Catalyst Support. Chem. Rev..

[22] Orlicka D., Simms N.J., Hussain T., Nicholls J.R. (2015). Comparison between Oxidation of Fe–Cr–Al Sputter Coatings in Air and Air–HCl Environments at 550 °C. Mater. High Temp..

[23] Zhu Z., Tan J., Wu X., Zhang Z., Han E.-H., Wang X. (2022). Corrosion Behaviors of FeCrAl Alloys Exposed to Oxygen-Saturated Static Lead Bismuth Eutectic at 550 °C. Corros. Sci..

[24] Zhang H., Ma J., Gao Z., Guo F., Xu S., Hou G., Zheng G. (2022). Study on Stability of Mechanical Properties for Porous Fe-Cr-Al Alloys after Long-Term Aging. Materials.

[25] Rakhadilov B., Kantay N., Sagdoldina Z., Erbolatuly D., Bektasova G., Paszkowski M. (2021). Experimental Investigations of Al_2_O_3_- and ZrO_2_-Based Coatings Deposited by Detonation Spraying. Mater. Res. Express.

[26] Lashmi P.G., Ananthapadmanabhan P.V., Unnikrishnan G., Aruna S.T. (2020). Present Status and Future Prospects of Plasma Sprayed Multilayered Thermal Barrier Coating Systems. J. Eur. Ceram. Soc..

[27] Rakhadilov B., Buitkenov D., Sagdoldina Z., Idrisheva Z., Zhamanbayeva M., Kakimzhanov D. (2021). Preparation and Characterization of NiCr/NiCr-Al_2_O_3_/Al_2_O_3_ Multilayer Gradient Coatings by Gas Detonation Spraying. Coatings.

[28] Wang P., Qiao Y., Qi W., Du S., Liu Z., Meng F., Zhang X., Wang K., Li Q., Yao Z. (2021). Preparation and Properties Study of Cr on FeCrAl Cladding Materials. Front. Mater..

[29] Rakhadilov B., Bayatanova L., Kengesbekov A., Magazov N., Toleukhanova Z., Yeskermessov D. (2025). Study of the Influence of Air Plasma Spraying Parameters on the Structure, Corrosion Resistance, and Tribological Characteristics of Fe–Al–Cr Intermetallic Coatings. Coatings.

[30] Zhong S., Chai L., Wang Z., Yang T., Liu Y., Dong H., Shen J., Li Y., Cheng E.J., Yin X. (2025). Microstructures and Tribological Properties of Laser-Clad FeCrAl-TiX Composite Coatings on Ferritic-Martensitic Steel. J. Mater. Res. Technol..

[31] Brezinová J., Landová M., Guzanová A., Dulebová Ľ., Draganovská D. (2018). Microstructure, Wear Behavior and Corrosion Resistance of WC-FeCrAl and WC-WB-Co Coatings. Metals.

